# Attitudes and Perceptions of Multidisciplinary Cancer Care Clinicians Toward Telehealth and Secure Messages

**DOI:** 10.1001/jamanetworkopen.2021.33877

**Published:** 2021-11-24

**Authors:** Elad Neeman, Deepika Kumar, Liisa Lyon, Tatjana Kolevska, Mary Reed, Tilak Sundaresan, Amit Arora, Yan Li, Samantha Seaward, Gillian Kuehner, Sharon Likely, Julia Trosman, Christine Weldon, Raymond Liu

**Affiliations:** 1 San Francisco Medical Center, Kaiser Permanente Northern California, San Francisco; 2 Division of Research, Kaiser Permanente Northern California, Oakland; 3 Napa/Solano Medical Center, Kaiser Permanente Northern California, Napa; 4 San Leandro Medical Center, Kaiser Permanente Northern California, San Leandro; 5 Oakland Medical Center, Kaiser Permanente Northern California, Oakland; 6 Richmond Medical Center, Kaiser Permanente Northern California, Richmond; 7 Vallejo Medical Center, Kaiser Permanente Northern California, Vallejo; 8 Modesto Medical Center, Kaiser Permanente Northern California, Modesto; 9 The Center for Business Models in Healthcare, Chicago, Illinois

## Abstract

**Importance:**

Telehealth use including secure messages has rapidly expanded since the COVID-19 pandemic, including for multidisciplinary aspects of cancer care. Recent reports described rapid uptake and various benefits for patients and clinicians, suggesting that telehealth may be in standard use after the pandemic.

**Objective:**

To examine attitudes and perceptions of multidisciplinary cancer care clinicians toward telehealth and secure messages.

**Design, Setting, and Participants:**

Cross-sectional specialty-specific survey (ie, some questions appear only for relevant specialties) among multidisciplinary cancer care clinicians, collected from April 29, 2020, to June 5, 2020. Participants were all 285 clinicians in the fields of medical oncology, radiation oncology, surgical oncology, survivorship, and oncology navigation from all 21 community cancer centers of Kaiser Permanente Northern California.

**Main Outcomes and Measures:**

Clinician satisfaction, perceived benefits and challenges of telehealth, perceived quality of telehealth and secure messaging, preferred visit and communication types for different clinical activities, and preferences regarding postpandemic telehealth use.

**Results:**

A total of 202 clinicians (71%) responded (104 of 128 medical oncologists, 34 of 37 radiation oncologists, 16 of 62 breast surgeons, 18 of 28 navigators, and 30 of 30 survivorship experts; 57% (116 of 202) were women; 73% [147 of 202] between ages 36-55 years). Seventy-six percent (n = 154) were satisfied with telehealth without statistically significant variations based on clinician characteristics. In-person visits were thought to promote a strong patient-clinician connection by 99% (n = 137) of respondents compared with 77% (n = 106) for video visits, 43% (n = 59) for telephone, and 14% (n = 19) for secure messages. The most commonly cited benefits of telehealth to clinicians included reduced commute (79%; n = 160), working from home (74%; n = 149), and staying on time (65%; n = 132); the most commonly cited negative factors included internet connection (84%; n = 170) or equipment problems (72%; n = 146), or physical examination needed (64%; n = 131). Most respondents (59%; n = 120) thought that video is adequate to manage the greater part of patient care in general; and most deemed various telehealth modalities suitable for any of the queried types of patient-clinician activities. For some specific activities, less than half of respondents thought that only an in-person visit is acceptable (eg, 49%; n = 66 for end-of-life discussion, 35%; n = 58 for new diagnosis). Most clinicians (82%; n = 166) preferred to maintain or increase use of telehealth after the pandemic.

**Conclusions and Relevance:**

In this survey of multidisciplinary cancer care clinicians in the COVID-19 era, telehealth was well received and often preferred by most cancer care clinicians, who deemed it appropriate to manage most aspects of cancer care. As telehealth use becomes routine in some cancer care settings, video and telephone visits and use of asynchronous secure messaging with patients in cancer care has clear potential to extend beyond the pandemic period.

## Key Points

**Question:**

What are the perceptions and experiences of multidisciplinary cancer care clinicians (medical oncologists, radiation oncologists, surgical oncologists, oncology navigators, and cancer survivorship) with regard to telehealth visits and exchanging secure messages with patients during the COVID-19 pandemic?

**Findings:**

In this survey study of 202 multidisciplinary cancer care clinicians, most were satisfied with telehealth and wished to maintain or increase future use. Despite being thought to promote a weaker patient-clinician connection, telehealth was considered appropriate by most to handle the greater part of patient assessment and care; however, some clinicians preferred in-person visits for certain activities.

**Meaning:**

Results of this survey study suggest that telehealth is well accepted by various cancer care clinicians, and is likely to remain used to some extent for most aspects of cancer care in the future.

## Teaser

This survey study assesses the attitudes and perceptions of multidisciplinary cancer care clinicians (from the fields of medical oncology, radiation oncology, surgical oncology, survivorship, and oncology navigation) toward telehealth and secure messages.

## Introduction

Telehealth, which includes digital and telecommunication tools such as telephone and video visits to manage patient care, as well as secure messages (ie,. emails via portal/app), have become more important during the current pandemic, given their potential role in reducing the spread of COVID-19. The Centers for Medicare & Medicaid Services has recently loosened restrictions on telehealth reimbursement, thus allowing its rapid uptake across different medical specialties.^1^ Telehealth has been shown to be comparable to in-person care in many clinical settings, with high satisfaction reported by both patients and clinicians.^2,3^ In cancer care, telehealth is now being used for remote chemotherapy supervision, symptom management, discussions regarding clinical trials, palliative care, survivorship care, and other activities.^2^ While published data on this matter are scarce, telehealth may potentially improve on-site capacity issues by reducing the need for physical rooms and support staff and allow social distancing in waiting and treatment areas. Kaiser Permanente Northern California (KPNC), which adopted telehealth well before the pandemic,^4^ has now rapidly expanded the use of telehealth-based services. Currently in this integrated health care system, most oncology clinician visits are conducted via video or telephone^5,6^

The rapid and sustained uptake of telehealth in oncology^6^ and other cancer care disciplines, as well as early indications of high satisfaction rates among patients with cancer and their clinicians,^7,8^ coupled with the various benefits for patients, caregivers, and clinicians,^9,10^ suggest that the use of telehealth may remain standard even after the COVID-19 pandemic has ended. Considering some of the potential concerns associated with telehealth,^8^ it is imperative that a clearer understanding of cancer care clinician attitudes and perceptions toward various components of telehealth be available as a vital step toward improving telehealth accessibility, quality, and safety.

Herein we study the attitudes and perceptions of multidisciplinary cancer care clinicians in the fields of medical oncology, radiation oncology, surgical oncology, survivorship, and oncology navigation toward telehealth and secure messages. We specifically focus on clinician satisfaction, perceived benefits and challenges of telehealth for clinicians, patients, and caregivers, perceived quality and effectiveness of various telehealth tools, preferred visit and communication types for different activities related to cancer care, and preferences regarding telehealth use after the pandemic.

## Methods

### Study Setting

We conducted a cross-sectional survey study among all multidisciplinary cancer care clinicians within KPNC. Clinicians included physicians (doctor of medicine or doctor of osteopathic medicine) specialized in medical oncology (n = 128), radiation oncology (n = 37), and breast surgery (n = 62), as well as nurse practitioners (n = 29) and physician assistants (n = 1) specialized in cancer survivorship, and other personnel (n = 28; registered nurses, social workers, health educators, and physician assistants) specialized in patient navigation. Data on race and ethnicity were not collected in this study as part of a survey of clinicians. KPNC is an integrated health care system serving more than 4.7 million members, with 262 medical offices, 21 hospitals, and 21 community cancer centers across the region. KPNC covers more than 30 percent of the population in the counties in which it has a physical presence, including the San Francisco Bay Area and the California Central Valley from Sacramento to Fresno. This study was reviewed by the Research Determination Official for the Kaiser Permanente Northern California region, which determined it to be exempt from ethics review because it did not meet the regulatory definition of research involving human subjects. This study followed the relevant portions of the Strengthening the Reporting of Observational Studies in Epidemiology (STROBE) reporting guideline^11^ for cross-sectional analysis and the American Association for Public Opinion Research (AAPOR) reporting guideline for survey studies.^12^

Under the integrated health care model at KPNC, all physicians and nonphysician clinicians receive a predetermined salary, and there is no difference in clinician compensation between telehealth and in-person patient encounters, regardless of how each patient is insured. Most members are insured by Kaiser Permanente directly, under an employer-based commercial plan, but some patients are insured through government plans such as Medicare Advantage or Medicaid. Telehealth services including telephone and video visits are billed specifically as such to the relevant insurance plan, but this billing does not affect the clinicians’ compensation. Many Kaiser Permanente insurance plans already eliminate or reduce patients’ out-of-pocket payment for telehealth visits, thus removing a potential barrier to telehealth for patients.^5^

Before the COVID-19 pandemic, KPNC had an established telehealth program, and physicians and patients could choose between office visits and video or telephone visits at their own discretion. Video visits in this system are conducted via a proprietary Health Insurance Portability and Accountability Act of 1996 (HIPAA)–compliant web portal or mobile app that allow for real-time language interpretation, as well as remote online visits if desired by the patient. As the first community-spread cases of COVID-19 were reported in California in March of 2020, KPNC decided to promote outpatient video (or, when not possible, telephone) telehealth visits, in all 21 KPNC cancer centers in lieu of in-person office visits whenever safe and feasible. KPNC clinicians were allowed and encouraged to work from home when performing telehealth services to further reduce infectious exposure risk. Because KPNC had an established though not consistently used^6^ telehealth program and secure message technology years before the pandemic,^4^ mechanisms for telehealth implementation were in place and the practice expanded rapidly.^6^ Specifically, before the COVID-19 pandemic, between January 1, 2019, and the California COVID-19-related shelter-in-place order on March 19, 2020, office visits comprised 65.4% of all hematology and oncology visits, telephone visits accounted for 31.7%, and the rest were video visits. In May 2020, when most survey responses were received, office visits accounted for 5.3% of visits, video visits for 30.6%, and telephone visits for 64.1% of all visits.^13^

### Data Collection

Between April 29, 2020, and May 14, 2020, emails were sent by 2 of us (T.K. and R.L.) to all KPNC medical oncologists, breast cancer surgeons, radiation oncologists, oncology patient navigators, and cancer survivorship clinicians. These clinicians were asked to fill a one-time specialty-specific voluntary electronic survey on their attitudes and perceptions regarding telehealth in cancer care. Surveys were specific to each specialty; some questions appeared only in surveys when those questions were deemed irrelevant to other clinical specialties. The surveys appear in the eMethods section in in the Supplement.

Review of the literature revealed no prior validated survey instruments relevant to the aims of this study. The primary creator of the surveys was the senior author (R.L.), but all surveys were created in collaboration with many co-authors, including medical oncologists (E.N., T.K., T.S., Y.L., A.A.), radiation oncologist (S.S.), breast surgeon (G.K.), business of health care experts (J.T., C.W.), nursing management expert (S.L.) and a KPNC Division of Research expert in telehealth and survey methodology research (M.R.). Feedback was solicited from leadership and other stakeholders from all participating specialties or departments. Patient or advocacy groups were not involved in development of these surveys. The first survey sent was for medical oncology on April 29, 2020; after receiving high response rates and interest from the other specialties, the additional specialty-specific surveys were sent within the subsequent 15 days. The surveys included questions from various domains: respondent characteristics, satisfaction with telehealth, benefits and challenges with telehealth, measures of perceived quality, and use cases for various forms of visits and patient communications. Up to 2 reminders were sent within the following month to nonresponders. All questions in the electronic surveys were multiple choice, most in Likert-scale form, with a required response, except for a few open-ended questions that were optional and are not reported herein due to low response rates. Microsoft Forms was used for composing surveys and collecting responses, and each clinician was allowed to fill the survey once. Survey responses were collected between April 29, 2020, and June 5, 2020.

### Statistical Analysis

Descriptive statistics were reported as proportions and frequencies. Bivariate comparisons of satisfaction with telehealth among various clinician groups or characteristics were performed using χ^2^ or Fisher exact test. For the bivariate comparisons, clinician satisfaction responses were grouped as either satisfied (very or somewhat) or not satisfied (neither satisfied nor unsatisfied, somewhat or very dissatisfied). Hypothesis tests were 2-sided. The significance threshold was set to *P* = .05, although no *P* values are reported because the results were not statistically significant for the statistical comparisons being made. Statistical analysis was completed using Microsoft Excel (Microsoft Corporation) and SAS statistical software version 9.4 (SAS Institute). Reported percentage values were rounded to the nearest whole number.

## Results

The online surveys were completed by 202 of 285 clinicians, which corresponds to an overall response rate of 71% (104 of 128 medical oncologists, 34 of 37 radiation oncologists, 16 of 62 of breast surgeons, 18 of 28 navigators, and 30 of 30 survivorship clinicians). There were no missing data in any of the reported results. Of the respondents, 57% (116 of 202) were female, 43% (84 of 202) were male, and 73% (147 of 202) were aged 36-55 years (Table 1).

**Table 1.** Self-reported Demographic and Study Characteristics of 202 Respondents

Characteristic	No. (%)
Sex	
Male	84 (43)
Female	116 (57)
Specialty	
Medical oncology	104 (51)
Radiation oncology	34 (17)
Breast surgery	16 (8)
Survivorship	30 (15)
Patient navigation	18 (9)
Age group, y	
<35	19 (9)
36-45	85 (42)
46-55	62 (31)
56-65	29 (14)
>65	6 (3)
Prefer not to say	1 (0)
Telehealth % of practice	
Prior to pandemic	
<25%	118 (58)
25%-50%	60 (30)
51%-75%	17 (8)
>75%	7 (3)
Since the pandemic	
<25%	2 (1)
25%-50%	13 (6)
51%-75%	28 (14)
>75%	159 (79)
Familiarity with telehealth technology	
Highly familiar	83 (41)
Somewhat familiar	98 (49)
Neither familiar nor unfamiliar	16 (8)
Somewhat familiar	4 (2)
Highly unfamiliar	1 (0)
% Time spent on clinical care	
<60%	18 (9)
60%-80%	156 (77)
>80%	28 (14)
Commute time prior to the pandemic (round trip), min/d	
<30	69 (34)
30-60	80 (40)
61-120	41 (20)
121-180	12 (6)
>180	0

**Figure 1.**
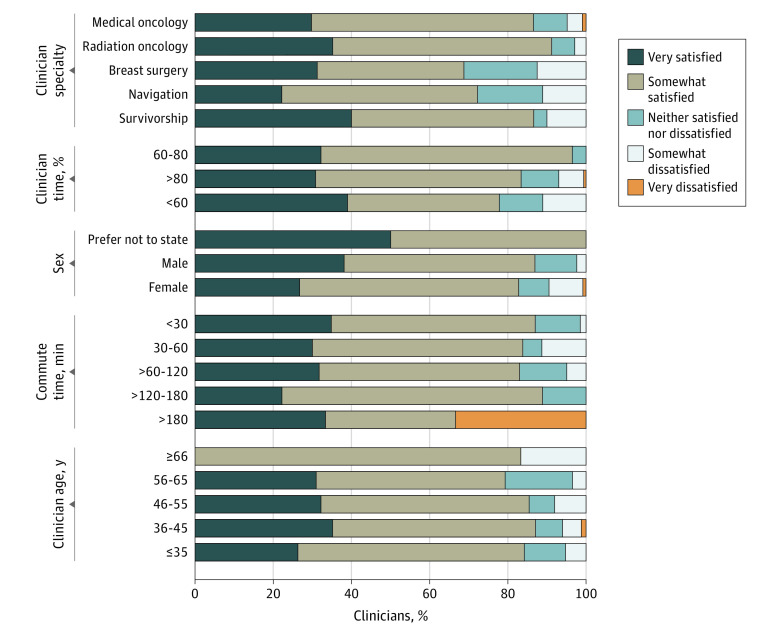
Clinician Satisfaction Levels With Telehealth

Overall, 76% (n = 154) of the respondents were either very satisfied or somewhat satisfied with telehealth, with the highest satisfaction rates among radiation oncologists (91% [31 of 34] either very satisfied or somewhat satisfied), and the lowest among breast surgeons (69% [11 of 16] either very satisfied or somewhat satisfied), but this difference was not statistically significant. There were additional modest differences in satisfaction with telehealth based on clinicians’ sex, age, self-reported familiarity with technology, percentage of clinical time, and commute time (Figure 1), but those differences were not statistically significant. When asked about other groups, most clinicians perceived satisfaction with telehealth among their patients (84% [169] thought their patients were highly or somewhat satisfied), patients’ caregivers (76% [154]), colleagues (81% [163]), and nonclinician staff (73% [147]). eFigure 1 in the Supplement provides full details.

Clinicians were asked what percentage of patient assessment and care can be managed by the various encounter types. Approximately half (51% [102 of 202]) of all clinicians thought more than 50% of patient assessment and care can be managed solely with in-person visits, 27% (n = 55) thought that only 25% to 50% of assessment and care can be managed solely with in-person visits, and 22% (n = 45) thought that less than 25% of all assessment and care can be managed by just in-person visits. Fifty nine percent (n = 120) responded that more than 50% of assessment and care can be managed by video visits; 33% (n = 67) felt this way regarding telephone visits and 4% (n = 9) felt this way about and secure messages (Table 2).

**Table 2.** Perceived Percentage of Patient Assessment and Care That Can Be Managed by In-Person, Video, Telephone, or Secure Messages

Encounter type by clinician specialty	No.	No. (%)			
		<25%	25%-50%	51%-75%	>75%
In person					
Medical oncology	104	26 (25)	31 (30)	10 (10)	37 (35)
Radiation oncology	34	6 (18)	8 (24)	7 (21)	13 (38)
Breast surgery	16	5 (31)	7 (44)	1 (6)	3 (19)
Navigation	18	3 (17)	3 (17)	5 (28)	7 (39)
Survivorship	30	5 (17)	6 (20)	7 (23)	12 (40)
Total	202	45 (22)	55 (27)	30 (15)	72 (36)
Video visits					
Medical oncology	104	4 (4)	35 (34)	48 (46)	17 (16)
Radiation oncology	34	2 (6)	10 (29)	14 (41)	8 (24)
Breast surgery	16	3 (19)	8 (50)	4 (25)	1 (6)
Navigation	18	3 (17)	5 (28)	9 (50)	1 (5)
Survivorship	30	2 (7)	10 (33)	13 (43)	5 (17)
Total	202	14 (7)	68 (33)	88 (44)	32 (16)
Telephone visits					
Medical oncology	104	8 (8)	61 (59)	29 (28)	6 (6)
Radiation oncology	34	2 (6)	20 (59)	10 (29)	2 (6)
Breast surgery	16	3 (19)	11 (69)	2 (12)	0
Navigation	18	0	7 (39)	10 (56)	1 (5)
Survivorship	30	5 (17)	18 (60)	4 (13)	3 (10)
Total	202	18 (9)	117 (58)	55 (27)	12 (6)
Secure messages					
Medical oncology	104	71 (68)	27 (26)	4 (4)	2 (2)
Radiation oncology	34	25 (74)	9 (26)	0	0
Breast surgery	16	12 (75)	4 (25)	0	0
Navigation	18	11 (61)	6 (33)	1 (6)	0
Survivorship	30	24 (80)	4 (13)	2 (7)	0
Total	202	143 (71)	50 (25)	7 (3)	2 (1)

Almost all clinicians (99% [137]) believed that in-person visits promote a strong clinician-patient connection compared with 77% (n = 106) who thought the same regarding video visits, and 43% (n = 59) for telephone visits. Only 14% (n = 19) responded that secure messages promote a strong clinician-patient connection (eFigure 2 in the Supplement).

**Figure 2.**
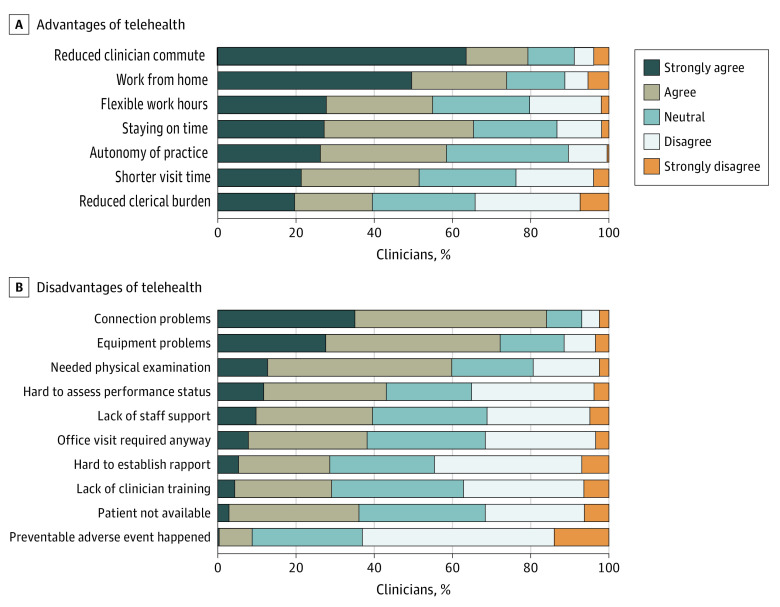
Clinician Perceptions of Benefits and Challenges With Telehealth

Benefits of telehealth most-commonly reported by clinicians included reduced commute (79%; n = 160 strongly agreed or agreed), working from home (74%; n = 149), and staying on time (65%; n = 132). The most commonly cited challenges with telehealth included connection problems (84%; n = 170 strongly agreed or agreed), equipment problems (72%; n = 146), a physical examination was required (60%; n = 131), difficulty in assessing performance status (43%; n = 87), and lack of staff support (40%; n = 80). Additional reported benefits and negative effects are shown in Figure 2.

**Figure 3.**
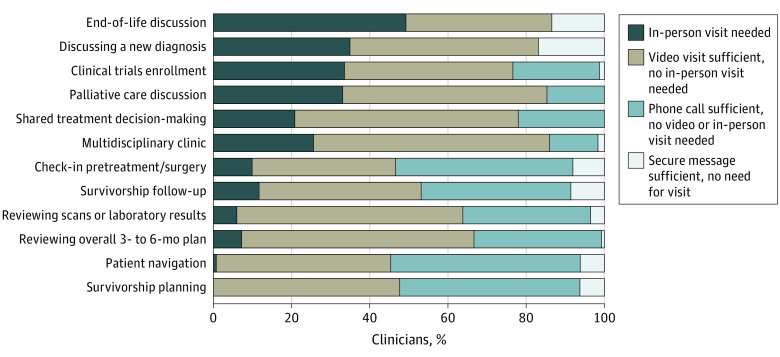
Encounter Types Deemed Most Appropriate by Clinicians for Various Cancer Care Related Activities

Telehealth modalities in total (combined video, telephone, and secure messages) were considered appropriate by most clinicians to address each of the various clinical activities related to cancer care that were included in our survey. For example, 49% (n = 66) of clinicians thought that only an in-person visit was acceptable for an end of life discussion, while 51% (n = 68) of respondents thought that a telehealth visit would suffice (specifically, 38% preferred video and 13% preferred a telephone visit for this activity). For discussing a new cancer diagnosis, 35% (n = 58) thought that an in-person visit was required, while 65% (n = 108) considered telehealth sufficient (48% video, 17% telephone). Additional types of activities for which some clinicians thought an in-person visit would be mandatory included clinical trial enrollment (34%; n = 53 of clinicians), palliative care discussion (33%; n = 45), multidisciplinary clinic (26%; n = 31), and making shared treatment decisions (21%; n = 35). Other clinical activities were even less commonly considered to require an in-person encounter (Figure 3). Telephone visits were deemed sufficient most commonly for patient navigation (49%; n = 63 of respondents), for survivorship planning (46%; n = 59), and check-in before treatment or surgery (45%; n = 73). Secure messages were rarely thought to be an appropriate method for any type of patient-clinician activity but were most acceptable for survivorship follow-up (9%; n = 11 of respondents), check-in pretreatment (8%; n = 13), and patient navigation (6%; n = 8) (Figure 3).

When asked about their wishes regarding the use of telehealth after the COVID-19 pandemic, most clinicians (82%; n = 86 of medical oncologists, 85%; n = 29 of radiation oncologists, 81%; n = 13 of breast surgeons, 61%; n = 11 of navigators, and 90%; n = 27 of survivorship clinicians) responded that they would either like to maintain or even increase rates of telehealth use. Only few respondents preferred to use less telehealth after the pandemic is over (6%; n = 6 of medical oncologists, 3%; n = 1 of radiation oncologists, 6%; n = 1; 6%; n = 1 of patient navigators, and 3%; n = 1 of survivorship clinicians (eFigure 3 in the Supplement).

## Discussion

Results of this survey study of multidisciplinary clinician attitudes and perceptions regarding the use of telehealth and secure messaging in cancer care during the COVID-19 pandemic highlight several notable findings. First, the results establish that in this health care system, most cancer care clinicians are satisfied with telehealth; this finding was consistent regardless of clinician specialty, age group, sex, commute time, familiarity with technology, and percentage of clinician time spent on clinical activities. Notably, because some of these groups included a small number of individuals, this lack of difference in satisfaction rates may be attributable to insufficient statistical power. Accordingly, most respondents of any of the participating specialties wished to maintain or increase the use of telehealth after the COVID-19 pandemic, and few preferred to reduce the usage of telehealth at that point. The responding clinicians also commonly perceived their patients, caregivers, colleagues, and staff to be highly satisfied with telehealth, with the patients being perceived as the most satisfied of those 4 groups. Other recent studies^8,14^ also reported high satisfaction with telehealth among patients with cancer.

Second, clinicians’ responses suggest that they deem telehealth to be equivalent or superior to in-person visits for many aspects of cancer care. For example, in-person visits were considered sufficient on their own for managing most patient assessment and care by only 51% of respondents, while 59% thought the same about video visits. However, clinician attitudes about the appropriateness of telehealth varied for different types of clinical activities related to cancer care. For example, most respondents thought that some activities, such as patient navigation, pretreatment visits, and survivorship visits can usually be handled by telephone or secure messaging, while other more serious activities, such as discussing a new diagnosis or end-of-life discussions, were thought to warrant an in-person or at least a video visit. These conclusions are consistent with a recently published abstract^15^ describing the outcomes of a National Cancer Center Network clinician survey, which reported that some activities, such as reviewing benign or reassuring data and follow-up for a patient receiving maintenance therapy or surveillance, are better handled with telehealth, while other activities, such as making decisions about therapy and establishing a personal connection with the patient, are better served by an in-person visit. Our study builds on this National Cancer Center Network survey in that it inquires about additional and other types of activities. This survey is also relevant to the more prevalent community cancer centers and not only academic NCCN-member institutions.

Our findings suggest that, overall, cancer care clinicians consider telehealth modalities as legitimate clinical tools integral to their practice that can be equivalent or even superior to in-person visits in many scenarios. This claim is further supported in that the most commonly cited telehealth-related benefits (eg, shorter commute, working from home, staying on time) and negative effects (connection and equipment problems) had to do with technical or nonclinical issues but not issues directly related to quality of care. Only a small fraction of respondents herein and elsewhere^15^ thought that an adverse event may have happened owing to telehealth use and could have been prevented with an in-person visit. Another concern about telehealth noted in this study was that various telehealth modalities were all considered inferior to in-person visits in establishing a patient-clinician connection.

### Strengths and Limitations

This study has important strengths: (1) we received high response rates from various clinician disciplines (except surgeons, which was similar to other prior studies^16,17^ in which there was a low response rate), that together encompass most aspects of current cancer care; (2) given that the KPNC health care system has years-long experience with the various telehealth modalities,^4^ it may be argued that the perceptions and attitudes reported herein are more mature and less affected by the novelty of telehealth technologies; (3) the KPNC patient population is large, highly diverse, and representative of the general population compared with most academic or single institutions; and (4) to our knowledge, this is one of the first studies on cancer care clinician attitudes toward secure messaging, which traditionally has not been included in telehealth studies but is gradually becoming a substantial component of primary^18^ and cancer care,^13^ and is desired and valued by patients.^19^

This study also has limitations. First, the scope of our study included only clinician attitudes and perceptions but not those of patients or caregivers. Future studies may focus on these populations. Second, given their socioeconomic status, the Northern Californian patient and clinician populations may likely be more familiar with the technologies used with telehealth (such as internet browsing and video conferencing) compared with populations in low-income countries and other parts of the US, where clinicians may have different experiences or attitudes toward telehealth. Third, as described in the Methods section, while the KPNC patient population is highly reflective of the communities it serves, KPNC provides health care through a capitated pay model in contrast to the fee-for-service payment model that is more common in the US.^20^ Given this situation, clinicians operating under noncapitated pay models may have different attitudes and perceptions of telehealth because using these services may affect their compensation levels. Fourth, our study period was relatively early in the COVID-19 pandemic era, and despite that in the KPNC health care system telehealth use did not change much in the subsequent months,^13^ clinician attitudes and perceptions regarding telehealth practices may have evolved. For example, clinicians may have initially performed their telehealth visits mostly from home and now may be performing more of these visits from the office, which would negate some benefits of working from home; however, this component was beyond the scope of this study. Fifth, our study was not powered to detect cross-specialty differences in attitudes and opinions toward telehealth, but such differences likely exist. For example, surgeons and radiation oncologists rely on physical presence to deliver important aspects of their care, whereas medical oncologists, survivorship experts, and navigators can deliver most of their care remotely. In addition, because of low interest by other surgical groups, we included only breast surgeons in this study. Future research may address these cross-specialty differences as they may affect the ongoing adoption and use of telehealth and secure messaging. In addition, telehealth services are currently not universally reimbursable by commercial and government insurance plans, which may reduce the ability of some practices to implement changes based on our findings.

The issue of payment parity for telehealth remains in determining the continued uptake and future use of telehealth, especially given the finding that most cancer care clinicians are interested in maintaining or increasing telehealth services after the pandemic. In the US, the Medicare Payment Advisory Commission recently recommended that policy makers extend current telehealth-related regulatory expansions for 1 to 2 more years after the pandemic to gather more evidence on the quality, access, and cost of telehealth, and inform permanent changes.^21^ Individual states^22,23^ are also promoting legislation to allow continued reimbursement for these services. These regulatory processes suggest that use of telehealth will likely remain a standard in the coming years.

## Conclusions

In this survey study of multidisciplinary cancer care clinicians, respondents reported high satisfaction from use of telehealth, perceived telehealth to be an integral tool essential to their practice, and in most cases considered various forms of telehealth to be appropriate for many types of patient care activities. Notably, most clinicians in each specialty preferred to maintain or increase levels of telehealth after the COVID-19 pandemic, and few preferred to reduce telehealth use. Collectively, these data suggest that to some extent telehealth will likely remain a key avenue of cancer care delivery in the future. As telehealth continues to take its place and become a recognized part of the hybrid model of cancer care, additional studies are warranted to confirm the quality and safety of these technologies, improve patient and clinician satisfaction, and explore and address disparities in patient and clinician access to telehealth.

## Notes

Supplement. **eFigure 1.** Clinicians’ Perceptions of Satisfaction With Telehealth Among Their Patients, Patient Caregivers, Colleagues, and Non-Clinician Staff **eFigure 2.** Clinicians’ Perceptions on the Strength of Patient-Clinician Connection Promoted by Various Encounter Types **eFigure 3.** Preferences of Different Clinician Types Regarding the Use of Telehealth After the COVID-19 Pandemic **eMethods.** Survey Instruments: Medical Oncology, Radiation Oncology, Breast Surgery, Patient Navigators, Survivorship
