# Supplementary material for: Attitudes and Perceptions of Multidisciplinary Cancer Care Clinicians Toward Telehealth and Secure Messages
Source: JAMA Netw Open. 2021 Nov 24;4(11):e2133877. doi: 10.1001/jamanetworkopen.2021.33877 (PMC8613601; doi:10.1001/jamanetworkopen.2021.33877)
Supplement: Supplement. — eFigure 1. Clinicians’ Perceptions of Satisfaction With Telehealth Among Their Patients, Patient Caregivers, Colleagues, and Non-Clinician Staff eFigure 2. Clinicians’ Perceptions on the Strength of Patient-Clinician Connection Promoted by Various Encounter Types eFigure 3. Preferences of Different Clinician Types Regarding the Use of Telehealth After the COVID-19 Pandemic eMethods. Survey Instruments: Medical Oncology, Radiation Oncology, Breast Surgery, Patient Navigators, Survivorship [file jamanetwopen-e2133877-s001.pdf]

Supplemental Online Content

Neeman E, Kumar D, Lyon L, et al. Attitudes and perceptions of multidisciplinary cancer care clinicians toward telehealth and secure messages. *JAMA Netw Open*. 2021;4(11):e2133877. doi:10.1001/jamanetworkopen.2021.33877

eFigure 1. Clinicians' Perceptions of Satisfaction With Telehealth Among Their Patients, Patient Caregivers, Colleagues, and Non-Clinician Staff

eFigure 2. Clinicians' Perceptions on the Strength of Patient-Clinician Connection Promoted by Various Encounter Types

eFigure 3. Preferences of Different Clinician Types Regarding the Use of Telehealth After the COVID-19 Pandemic

eMethods. Survey Instruments: Medical Oncology, Radiation Oncology, Breast Surgery, Patient Navigators, Survivorship

This supplemental material has been provided by the authors to give readers additional information about their work.

© 2021 Neeman E et al. *JAMA Network Open.*

1. eFigure 1: Clinicians' perceptions of satisfaction with telehealth among their patients, patient caregivers, colleagues, and non-clinician staff

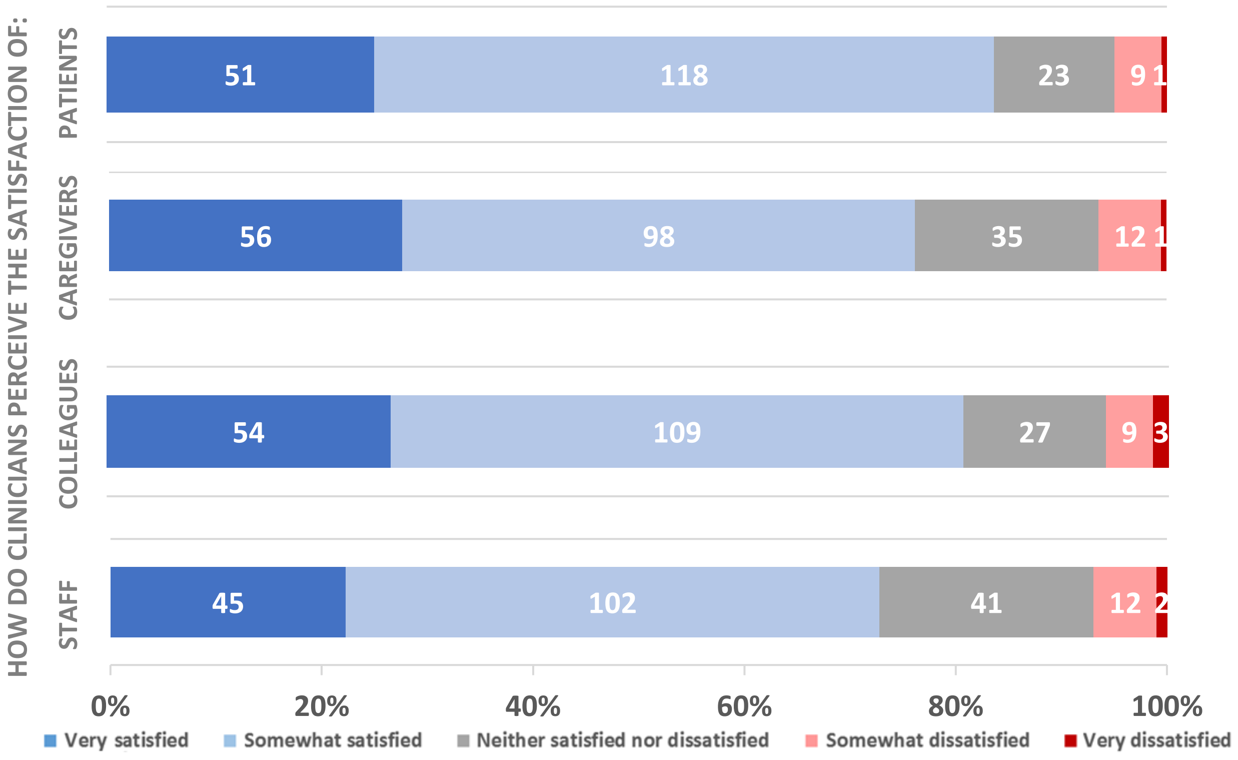

© 2021 Neeman E et al. *JAMA Network Open.*

2. eFigure 2: Clinicians' perceptions on the strength of patient-clinician connection promoted by various encounter types

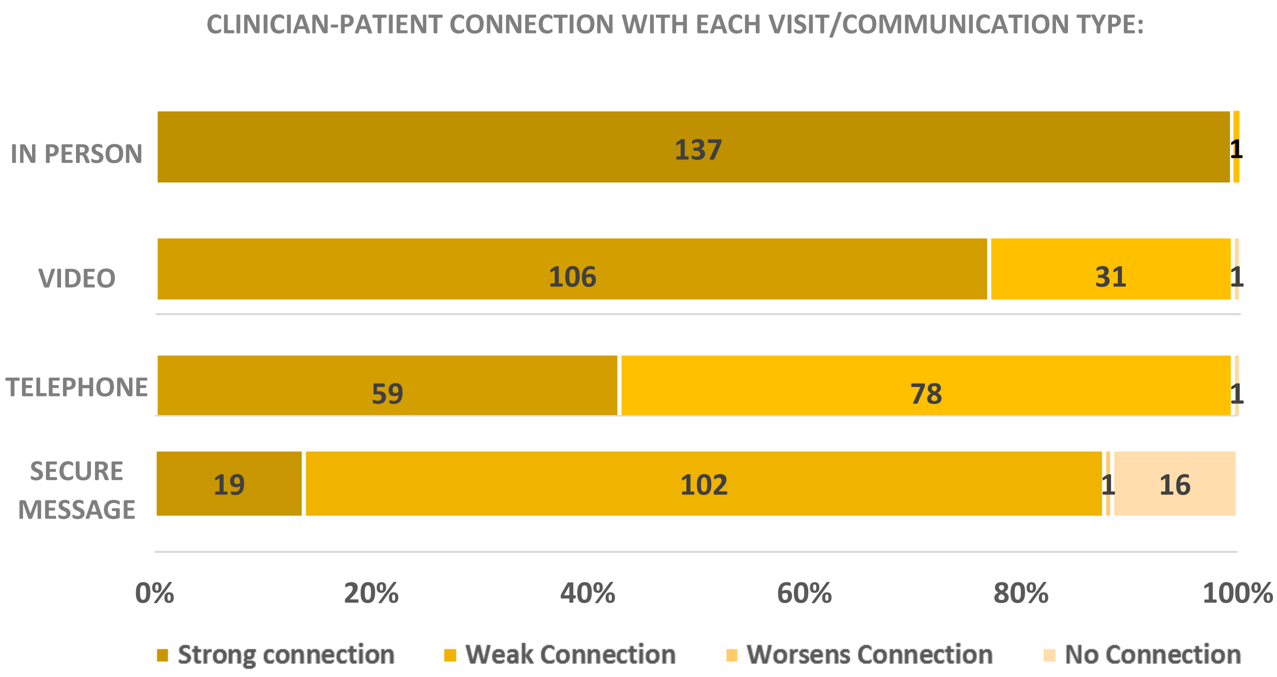

© 2021 Neeman E et al. *JAMA Network Open.*

3. eFigure 3: Preferences of different clinician types regarding the use of telehealth after the COVID-19 pandemic

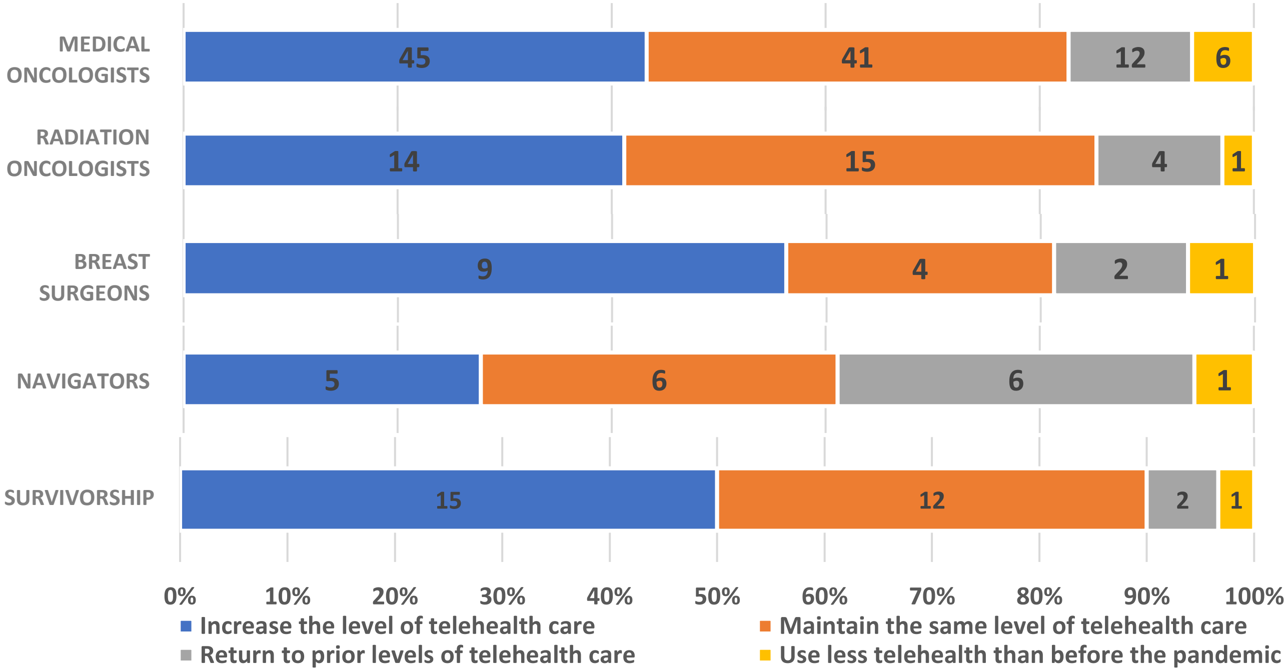

© 2021 Neeman E et al. *JAMA Network Open.*

4. Survey tool: Medical Oncology

MD/DO Provider Survey Telehealth Oncology

Thank you for helping us improve telehealth for our team, our colleagues, and our patients and their families.
Information will be reported out in aggregate, anonymous format only.

Telehealth is defined for the purposes of this study to include email, telephone, and video visits.

* Required

* This form will record your name, please fill your name.

1. What % of your patient encounters are related to telehealth (email, telephone, video)? *

	<25%	26-50%	51-75%	>75%
Before pandemic started	○	○	○	○
During pandemic	○	○	○	○
Expected, after pandemic is over	○	○	○	○

© 2021 Neeman E et al. *JAMA Network Open.*

2. For each of the activities below, please select the method that you believe could be used for a discussion with a patient: *

	Email sufficient, nothing else needed	Phone call sufficient, no video or in person needed	Video visit sufficient, no in person needed	Video visit with shared screen sufficient, no in person needed	In person needed	N/A - I don't preform these types of visits
Discussing a new patient diagnosis	○	○	○	○	○	○
Shared decision making / treatment decisions	○	○	○	○	○	○
Discussing scans / test results	○	○	○	○	○	○
Check in pre-treatment	○	○	○	○	○	○
Palliative care discussion	○	○	○	○	○	○
End of life discussion	○	○	○	○	○	○
Survivorship planning	○	○	○	○	○	○
Survivorship follow-up	○	○	○	○	○	○
Multidisciplinary clinic	○	○	○	○	○	○
Discussion of overall care plan for the next 3-6 months (e.g., sequence of surgery, chemotherapy, radiation, supportive care, etc.)	○	○	○	○	○	○
Patient navigation	○	○	○	○	○	○
Clinical trials enrollment	○	○	○	○	○	○
Clinical trials follow-up	○	○	○	○	○	○

© 2021 Neeman E et al. *JAMA Network Open.*

3. Are there other visits that could be performed using telehealth, or visits that could not be performed by telehealth? (optional)

4. After COVID-19 is no longer an active issue, would you prefer to: *

- ◯ Increase the level of telehealth care
- ◯ Maintain the same level of telehealth care
- ◯ Return to prior levels of telehealth care
- ◯ Use less telehealth than before the pandemic

5. Why (Optional)?

6. What % of patient assessment and care can be managed by the following methods: *

	<25%	25-50%	51-75%	76%-100%
Email	◯	◯	◯	◯
Telephone	◯	◯	◯	◯
Video	◯	◯	◯	◯
In person visit	◯	◯	◯	◯

© 2021 Neeman E et al. *JAMA Network Open.*

9. Which of the following benefits of telehealth have your patients or their caregivers experienced? *

	Strongly agree	Agree	Neutral	Disagree	Strongly disagree
Quicker attention to issue or concern	○	○	○	○	○
Less financial burden (copays, parking, etc.)	○	○	○	○	○
More desirable appointment times	○	○	○	○	○
Convenience for caregivers (time off work, etc.)	○	○	○	○	○
More connection with care team or provider	○	○	○	○	○
Patients able to discuss sensitive situations that would be embarrassing or difficult to address in person	○	○	○	○	○

© 2021 Neeman E et al. *JAMA Network Open.*

10. Which negative effects of telehealth have you personally experienced? *

	Strongly agree	Agree	Neutral	Disagree	Strongly disagree
Equipment problems	○	○	○	○	○
Lack of provider training	○	○	○	○	○
Connection problems	○	○	○	○	○
Lack of staff support	○	○	○	○	○
Lack of staff training	○	○	○	○	○
Patient issue should have been answered by another member of the care team	○	○	○	○	○
In person visit required anyway	○	○	○	○	○
Needed physical exam	○	○	○	○	○
Difficulty evaluating performance status	○	○	○	○	○
Difficulty establishing rapport	○	○	○	○	○
Patient suffered an adverse effect that could have been prevented with in-person visit	○	○	○	○	○
Patient not available	○	○	○	○	○

11. What are other benefits or downsides of telemedicine that have not been listed? (optional)

© 2021 Neeman E et al. *JAMA Network Open.*

12. Rate the level of provider-patient connection that each method of communication provides: *

	Strong connection	Weak Connection	No Connection	Worsens Connection
E-mail	○	○	○	○
Telephone	○	○	○	○
Video	○	○	○	○
In person	○	○	○	○

© 2021 Neeman E et al. *JAMA Network Open.*

13. Describe your experience with video visits: *

	Strongly Agree	Agree	Neutral	Disagree	Strongly Disagree
Video visits are easy to implement into my daily clinic work-flow	○	○	○	○	○
I prefer video visits to in-person visits	○	○	○	○	○
I prefer video visits to telephone visits	○	○	○	○	○
I prefer video visits to emails	○	○	○	○	○
Video visits reduce my clerical burden	○	○	○	○	○
I would recommend video visits to other providers	○	○	○	○	○
My patients' concerns are addressed by video visits	○	○	○	○	○
The quality of care provided through video visits is nearly equivalent to in-person visits	○	○	○	○	○
My patients have had technical challenges with using video visits	○	○	○	○	○

14. Anything else about video visits that you would like to share? (optional)

© 2021 Neeman E et al. *JAMA Network Open.*

15. Rate yourself on your familiarity with technology: *

	Highly familiar	Somewhat familiar	Neither familiar or unfamiliar	Somewhat unfamiliar	Highly unfamiliar
Email	◯	◯	◯	◯	◯
Telephone	◯	◯	◯	◯	◯
Video	◯	◯	◯	◯	◯
Electronic Medical Record	◯	◯	◯	◯	◯
General tech savviness	◯	◯	◯	◯	◯

16. Please indicate any additional telehealth training you may need and enter your contact information if we can contact you re: the training (optional)

17. What is your age? *

◯ 35 or under

◯ 36-45

◯ 46-55

◯ 56-65

◯ 66 and older

◯ Prefer Not to State

© 2021 Neeman E et al. *JAMA Network Open.*

18. What is your gender? *

- ◯ Female
- ◯ Male
- ◯ Prefer Not to State
- ◯ Other

19. What % of your time is spent on clinical care? *

- ◯ < 60%
- ◯ 60-80%
- ◯ > 80%

20. What was your commute time prior to the pandemic (minutes a day, round trip) *

- ◯ < 30 minutes
- ◯ 30 minutes - 60 minutes
- ◯ >60 minutes - 120 minutes
- ◯ >120 minutes - 180 minutes
- ◯ >180 minutes

© 2021 Neeman E et al. *JAMA Network Open.*

5. Survey tool: Radiation Oncology

Radiation Oncology Survey Telehealth Oncology

Thank you for helping us improve telehealth for our team, our colleagues, and our patients and their families.
Information will be reported out in aggregate, anonymous format only.

Telehealth is defined for the purposes of this study to include email, telephone, and video visits.

* Required

* This form will record your name, please fill your name.

1. What % of your patient encounters are related to telehealth (email, telephone, video)? *

	<25%	26-50%	51-75%	>75%
Before pandemic started	○	○	○	○
During pandemic	○	○	○	○
Expected, after pandemic is over	○	○	○	○

© 2021 Neeman E et al. *JAMA Network Open.*

2. For each of the activities below, please select the method that you believe could be used for a discussion with a patient: *

	Email sufficient, nothing else needed	Phone call sufficient, no video or in person needed	Video visit sufficient, no in person needed	Video visit with shared screen sufficient, no in person needed	In person needed	N/A - I don't preform these types of visits
Discussing a new patient diagnosis	◯	◯	◯	◯	◯	◯
Shared decision making / treatment decisions	◯	◯	◯	◯	◯	◯
Discussing scans / test results	◯	◯	◯	◯	◯	◯
Check in pre-treatment	◯	◯	◯	◯	◯	◯
Palliative care discussion	◯	◯	◯	◯	◯	◯
End of life discussion	◯	◯	◯	◯	◯	◯
Survivorship planning	◯	◯	◯	◯	◯	◯
Survivorship follow-up	◯	◯	◯	◯	◯	◯
Multidisciplinary clinic	◯	◯	◯	◯	◯	◯
Discussion of overall care plan for the next 3-6 months (e.g., sequence of surgery, chemotherapy, radiation, supportive care, etc.)	◯	◯	◯	◯	◯	◯
Patient navigation	◯	◯	◯	◯	◯	◯
Clinical trials enrollment	◯	◯	◯	◯	◯	◯
Clinical trials follow-up	◯	◯	◯	◯	◯	◯

© 2021 Neeman E et al. *JAMA Network Open.*

3. Are there other visits that could be performed using telehealth, or visits that could not be performed by telehealth? (optional)

4. After COVID-19 is no longer an active issue, would you prefer to: *

- ◯ Increase the level of telehealth care
- ◯ Maintain the same level of telehealth care
- ◯ Return to prior levels of telehealth care
- ◯ Use less telehealth than before the pandemic

5. Why (Optional)?

6. What % of patient assessment and care can be managed by the following methods: *

	<25%	25-50%	51-75%	76%-100%
Email	◯	◯	◯	◯
Telephone	◯	◯	◯	◯
Video	◯	◯	◯	◯
In person visit	◯	◯	◯	◯

© 2021 Neeman E et al. *JAMA Network Open.*

7. In your opinion, what is the satisfaction level with telehealth: *

	Very Satisfied	Somewhat satisfied	Neither satisfied nor dissatisfied	Somewhat dissatisfied	Very dissatisfied
Your satisfaction	◯	◯	◯	◯	◯
Colleague satisfaction	◯	◯	◯	◯	◯
Staff satisfaction	◯	◯	◯	◯	◯
Patient satisfaction	◯	◯	◯	◯	◯
Caregiver satisfaction	◯	◯	◯	◯	◯

8. Which of the following benefits of telehealth have you personally experienced? *

	Strong agree	Agree	Neutral	Disagree	Strongly Disagree
Work from home	◯	◯	◯	◯	◯
Reduced commute	◯	◯	◯	◯	◯
Shorter visit time	◯	◯	◯	◯	◯
Stay on time	◯	◯	◯	◯	◯
Autonomy of practice	◯	◯	◯	◯	◯
Flexible hours	◯	◯	◯	◯	◯
Reduced clerical burden	◯	◯	◯	◯	◯

© 2021 Neeman E et al. *JAMA Network Open.*

9. Which of the following benefits of telehealth have your patients or their caregivers experienced? *

	Strongly agree	Agree	Neutral	Disagree	Strongly disagree
Quicker attention to issue or concern	○	○	○	○	○
Less financial burden (copays, parking, etc.)	○	○	○	○	○
More desirable appointment times	○	○	○	○	○
Convenience for caregivers (time off work, etc.)	○	○	○	○	○
More connection with care team or provider	○	○	○	○	○
Patients able to discuss sensitive situations that would be embarrassing or difficult to address in person	○	○	○	○	○

© 2021 Neeman E et al. *JAMA Network Open.*

10. Which negative effects of telehealth have you personally experienced? *

	Strongly agree	Agree	Neutral	Disagree	Strongly disagree
Equipment problems	◯	◯	◯	◯	◯
Lack of provider training	◯	◯	◯	◯	◯
Connection problems	◯	◯	◯	◯	◯
Lack of staff support	◯	◯	◯	◯	◯
Lack of staff training	◯	◯	◯	◯	◯
Patient issue should have been answered by another member of the care team	◯	◯	◯	◯	◯
In person visit required anyway	◯	◯	◯	◯	◯
Needed physical exam	◯	◯	◯	◯	◯
Difficulty evaluating performance status	◯	◯	◯	◯	◯
Difficulty establishing rapport	◯	◯	◯	◯	◯
Patient suffered an adverse effect that could have been prevented with in-person visit	◯	◯	◯	◯	◯
Patient not available	◯	◯	◯	◯	◯

11. What are other benefits or downsides of telemedicine that have not been listed? (optional)

© 2021 Neeman E et al. *JAMA Network Open.*

12. Rate the level of provider-patient connection that each method of communication provides: *

	Strong connection	Weak Connection	No Connection	Worsens Connection
E-mail	○	○	○	○
Telephone	○	○	○	○
Video	○	○	○	○
In person	○	○	○	○

© 2021 Neeman E et al. *JAMA Network Open.*

13. Describe your experience with video visits: *

	Strongly Agree	Agree	Neutral	Disagree	Strongly Disagree
Video visits are easy to implement into my daily clinic work-flow	○	○	○	○	○
I prefer video visits to in-person visits	○	○	○	○	○
I prefer video visits to telephone visits	○	○	○	○	○
I prefer video visits to emails	○	○	○	○	○
Video visits reduce my clerical burden	○	○	○	○	○
I would recommend video visits to other providers	○	○	○	○	○
My patients' concerns are addressed by video visits	○	○	○	○	○
The quality of care provided through video visits is nearly equivalent to in-person visits	○	○	○	○	○
My patients have had technical challenges with using video visits	○	○	○	○	○

14. Anything else about video visits that you would like to share? (optional)

© 2021 Neeman E et al. *JAMA Network Open.*

15. Rate yourself on your familiarity with technology: *

	Highly familiar	Somewhat familiar	Neither familiar or unfamiliar	Somewhat unfamiliar	Highly unfamiliar
Email	○	○	○	○	○
Telephone	○	○	○	○	○
Video	○	○	○	○	○
Electronic Medical Record	○	○	○	○	○
General tech savviness	○	○	○	○	○

16. Please indicate any additional telehealth training you may need and enter your contact information if we can contact you re: the training (optional)

17. What is your age? *

- ○ 35 or under
- ○ 36-45
- ○ 46-55
- ○ 56-65
- ○ 66 and older
- ○ Prefer Not to State

© 2021 Neeman E et al. *JAMA Network Open.*

18. What is your gender? *

- ◯ Female
- ◯ Male
- ◯ Prefer Not to State
- ◯ Other

19. What % of your time is spent on clinical care? *

- ◯ < 60%
- ◯ 60-80%
- ◯ > 80%

20. What was your commute time prior to the pandemic (minutes a day, round trip) *

- ◯ < 30 minutes
- ◯ 30 minutes - 60 minutes
- ◯ >60 minutes - 120 minutes
- ◯ >120 minutes - 180 minutes
- ◯ >180 minutes

© 2021 Neeman E et al. *JAMA Network Open.*

6. Survey tool: Breast Surgery

Breast Cancer Surgery Survey Telehealth

Thank you for helping us improve telehealth for our team, our colleagues, and our patients and their families.
Information will be reported out in aggregate, anonymous format only.

Telehealth is defined for the purposes of this study to include email, telephone, and video visits.

* Required

* This form will record your name, please fill your name.

1. What portion of your practice involves breast cancer patients? *

- ◯ None
- ◯ <25%
- ◯ 26-50%
- ◯ 51-75%
- ◯ >75%

2. What % of your breast cancer patient encounters are related to telehealth (email, telephone, video)? *

	<25%	26-50%	51-75%	>75%
Before pandemic started	◯	◯	◯	◯
During pandemic	◯	◯	◯	◯
Expected, after pandemic is over	◯	◯	◯	◯

© 2021 Neeman E et al. *JAMA Network Open.*

3. For each of the activities below, please select the method that you believe could be used for a discussion with a breast cancer patient: *

	Email sufficient, nothing else needed	Phone call sufficient, no video or in person needed	Video visit sufficient, no in person needed	Video visit with shared screen sufficient, no in person needed	In person needed	N/A - I don't preform these types of visits
Discussing a new diagnosis	◯	◯	◯	◯	◯	◯
Shared decision making and surgical consultation/planning	◯	◯	◯	◯	◯	◯
Discussing scans / test results	◯	◯	◯	◯	◯	◯
Check in pre-surgery	◯	◯	◯	◯	◯	◯
Postoperative visit	◯	◯	◯	◯	◯	◯
Discussion of overall care plan for the next 3-6 months (e.g., sequence of surgery, chemotherapy, radiation, supportive care, etc.)	◯	◯	◯	◯	◯	◯
Clinical trials enrollment	◯	◯	◯	◯	◯	◯

4. Are there other visits that could be performed using telehealth, or visits that could not be performed by telehealth? (optional)

© 2021 Neeman E et al. *JAMA Network Open.*

5. After COVID-19 is no longer an active issue, would you prefer to: *

- ◯ Increase the level of telehealth care
- ◯ Maintain the same level of telehealth care
- ◯ Return to prior levels of telehealth care
- ◯ Use less telehealth than before the pandemic

6. Why (Optional)?

[ ]

7. What % of breast cancer patient assessment and care can be managed by the following methods: *

	<25%	25-50%	51-75%	76%-100%
Email	◯	◯	◯	◯
Telephone	◯	◯	◯	◯
Video	◯	◯	◯	◯
In person visit	◯	◯	◯	◯

© 2021 Neeman E et al. *JAMA Network Open.*

8. In your opinion, what is the satisfaction level with telehealth: *

	Very Satisfied	Somewhat satisfied	Neither satisfied nor dissatisfied	Somewhat dissatisfied	Very dissatisfied
Your satisfaction	○	○	○	○	○
Colleague satisfaction	○	○	○	○	○
Staff satisfaction	○	○	○	○	○
Patient satisfaction	○	○	○	○	○
Caregiver satisfaction	○	○	○	○	○

9. Which of the following benefits of telehealth have you personally experienced? *

	Strong agree	Agree	Neutral	Disagree	Strongly Disagree
Work from home	○	○	○	○	○
Reduced commute	○	○	○	○	○
Shorter visit time	○	○	○	○	○
Stay on time	○	○	○	○	○
Autonomy of practice	○	○	○	○	○
Flexible hours	○	○	○	○	○
Reduced clerical burden	○	○	○	○	○

© 2021 Neeman E et al. *JAMA Network Open.*

10. Which of the following benefits of telehealth have your breast cancer patients or their caregivers experienced? *

	Strongly agree	Agree	Neutral	Disagree	Strongly disagree
Quicker attention to issue or concern	○	○	○	○	○
Less financial burden (copays, parking, etc.)	○	○	○	○	○
More desirable appointment times	○	○	○	○	○
Convenience for caregivers (time off work, etc.)	○	○	○	○	○
More connection with care team or provider	○	○	○	○	○
Patients able to discuss sensitive situations that would be embarrassing or difficult to address in person	○	○	○	○	○

© 2021 Neeman E et al. *JAMA Network Open.*

11. Which negative effects of telehealth have you personally experienced? *

	Strongly agree	Agree	Neutral	Disagree	Strongly disagree
Equipment problems	○	○	○	○	○
Lack of provider training	○	○	○	○	○
Connection problems	○	○	○	○	○
Lack of staff support	○	○	○	○	○
Lack of staff training	○	○	○	○	○
Patient issue should have been answered by another member of the care team	○	○	○	○	○
In person visit required anyway	○	○	○	○	○
Needed physical exam or photos	○	○	○	○	○
Difficulty evaluating performance status	○	○	○	○	○
Difficulty establishing rapport	○	○	○	○	○
Patient suffered an adverse effect that could have been prevented with in-person visit	○	○	○	○	○
Patient not available	○	○	○	○	○

© 2021 Neeman E et al. *JAMA Network Open.*

12. What are other benefits or downsides of telemedicine that have not been listed? (optional)

13. Rate the level of provider-patient connection that each method of communication provides: *

	Strong connection	Weak Connection	No Connection	Worsens Connection
E-mail	○	○	○	○
Telephone	○	○	○	○
Video	○	○	○	○
In person	○	○	○	○

© 2021 Neeman E et al. *JAMA Network Open.*

14. Describe your experience with video visits: *

	Strongly Agree	Agree	Neutral	Disagree	Strongly Disagree
Video visits are easy to implement into my daily clinic work-flow	○	○	○	○	○
I prefer video visits to in-person visits	○	○	○	○	○
I prefer video visits to telephone visits	○	○	○	○	○
I prefer video visits to emails	○	○	○	○	○
Video visits reduce my clerical burden	○	○	○	○	○
I would recommend video visits to other providers	○	○	○	○	○
My patients' concerns are addressed by video visits	○	○	○	○	○
The quality of care provided through video visits is nearly equivalent to in-person visits	○	○	○	○	○
My patients have had technical challenges with using video visits	○	○	○	○	○

15. Anything else about video visits that you would like to share? (optional)

© 2021 Neeman E et al. *JAMA Network Open.*

16. Rate yourself on your familiarity with technology: *

	Highly familiar	Somewhat familiar	Neither familiar or unfamiliar	Somewhat unfamiliar	Highly unfamiliar
Email	○	○	○	○	○
Telephone	○	○	○	○	○
Video	○	○	○	○	○
Electronic Medical Record	○	○	○	○	○
General tech savviness	○	○	○	○	○

17. Please indicate any additional telehealth training you may need and enter your contact information if we can contact you re: the training (optional)

18. What is your age? *

- ○ 35 or under
- ○ 36-45
- ○ 46-55
- ○ 56-65
- ○ 66 and older
- ○ Prefer Not to State

© 2021 Neeman E et al. *JAMA Network Open.*

19. What is your gender? *

- ◯ Female
- ◯ Male
- ◯ Prefer Not to State
- ◯ Other

20. What % of your time is spent on clinical care? *

- ◯ < 60%
- ◯ 60-80%
- ◯ > 80%

21. What was your commute time prior to the pandemic (minutes a day, round trip) *

- ◯ < 30 minutes
- ◯ 30 minutes - 60 minutes
- ◯ >60 minutes - 120 minutes
- ◯ >120 minutes - 180 minutes
- ◯ >180 minutes

© 2021 Neeman E et al. *JAMA Network Open.*

7. Survey tool: Patient Navigators

Navigator Survey Telehealth Oncology

Thank you for helping us improve telehealth for our team, our colleagues, and our patients and their families.
Information will be reported out in aggregate, anonymous format only.

Telehealth is defined for the purposes of this study to include email, telephone, and video visits.

* Required

* This form will record your name, please fill your name.

[ ]

1. In which department are you located? *

- ◯ Medical Oncology
- ◯ Breast Surgery
- ◯ Radiology
- ◯ [ ] Other

© 2021 Neeman E et al. *JAMA Network Open.*

2. What is your certification? *

- ◯ RN
- ◯ LCSW
- ◯ Health educator
- ◯ NP
- ◯ PA
- ◯ Other

3. What % of your patient encounters are related to telehealth (email, telephone, video)? *

	<25%	26-50%	51-75%	>75%
Before pandemic started	◯	◯	◯	◯
During pandemic	◯	◯	◯	◯
Expected, after pandemic is over	◯	◯	◯	◯

© 2021 Neeman E et al. *JAMA Network Open.*

4. For each of the activities below, please select the method that you believe could be used for a discussion with a patient: *

	Email sufficient, nothing else needed	Phone call sufficient, no video or in person needed	Video visit sufficient, no in person needed	Video visit with shared screen sufficient, no in person needed	In person needed	N/A - I don't preform these types of visits
Discussing a new patient diagnosis						
Shared decision making / treatment decisions						
Discussing scans / test results						
Check in pre-treatment						
Palliative care discussion						
Survivorship planning						
Survivorship follow-up						
Multidisciplinary clinic						
Discussion of overall care plan for the next 3-6 months (e.g., sequence of surgery, chemotherapy, radiation, supportive care, etc.)						
Patient navigation						
Clinical trials enrollment						
Clinical trials follow-up						

© 2021 Neeman E et al. *JAMA Network Open.*

5. Are there other visits that could be performed using telehealth, or visits that could not be performed by telehealth? (optional)

6. After COVID-19 is no longer an active issue, would you prefer to: *

- ◯ Increase the level of telehealth care
- ◯ Maintain the same level of telehealth care
- ◯ Return to prior levels of telehealth care
- ◯ Use less telehealth than before the pandemic

7. Why (Optional)?

8. What % of patient assessment and care can be managed by the following methods: *

	<25%	25-50%	51-75%	76%-100%
Email	◯	◯	◯	◯
Telephone	◯	◯	◯	◯
Video	◯	◯	◯	◯
In person visit	◯	◯	◯	◯

© 2021 Neeman E et al. *JAMA Network Open.*

9. In your opinion, what is the satisfaction level with telehealth: *

	Very Satisfied	Somewhat satisfied	Neither satisfied nor dissatisfied	Somewhat dissatisfied	Very dissatisfied
Your satisfaction	◯	◯	◯	◯	◯
Colleague satisfaction	◯	◯	◯	◯	◯
Staff satisfaction	◯	◯	◯	◯	◯
Patient satisfaction	◯	◯	◯	◯	◯
Caregiver satisfaction	◯	◯	◯	◯	◯

10. Which of the following benefits of telehealth have you personally experienced? *

	Strong agree	Agree	Neutral	Disagree	Strongly Disagree
Work from home	◯	◯	◯	◯	◯
Reduced commute	◯	◯	◯	◯	◯
Shorter visit time	◯	◯	◯	◯	◯
Stay on time	◯	◯	◯	◯	◯
Autonomy of practice	◯	◯	◯	◯	◯
Flexible hours	◯	◯	◯	◯	◯
Reduced clerical burden	◯	◯	◯	◯	◯

© 2021 Neeman E et al. *JAMA Network Open.*

11. Which of the following benefits of telehealth have your patients or their caregivers experienced? *

	Strongly agree	Agree	Neutral	Disagree	Strongly disagree
Quicker attention to issue or concern	○	○	○	○	○
Less financial burden (copays, parking, etc.)	○	○	○	○	○
More desirable appointment times	○	○	○	○	○
Convenience for caregivers (time off work, etc.)	○	○	○	○	○
More connection with care team or provider	○	○	○	○	○
Patients able to discuss sensitive situations that would be embarrassing or difficult to address in person	○	○	○	○	○

© 2021 Neeman E et al. *JAMA Network Open.*

12. Which negative effects of telehealth have you personally experienced? *

	Strongly agree	Agree	Neutral	Disagree	Strongly disagree
Equipment problems	○	○	○	○	○
Lack of provider training	○	○	○	○	○
Connection problems	○	○	○	○	○
Lack of staff support	○	○	○	○	○
Lack of staff training	○	○	○	○	○
Patient issue should have been answered by another member of the care team	○	○	○	○	○
In person visit required anyway	○	○	○	○	○
Needed physical exam	○	○	○	○	○
Difficulty evaluating performance status	○	○	○	○	○
Difficulty establishing rapport	○	○	○	○	○
Patient suffered an adverse effect that could have been prevented with in-person visit	○	○	○	○	○
Patient not available	○	○	○	○	○

13. What are other benefits or downsides of telemedicine that have not been listed? (optional)

© 2021 Neeman E et al. *JAMA Network Open.*

14. Rate the level of provider-patient connection that each method of communication provides: *

	Strong connection	Weak Connection	No Connection	Worsens Connection
E-mail	○	○	○	○
Telephone	○	○	○	○
Video	○	○	○	○
In person	○	○	○	○

© 2021 Neeman E et al. *JAMA Network Open.*

15. Describe your experience with video visits: *

	Strongly Agree	Agree	Neutral	Disagree	Strongly Disagree
Video visits are easy to implement into my daily clinic work-flow	○	○	○	○	○
I prefer video visits to in-person visits	○	○	○	○	○
I prefer video visits to telephone visits	○	○	○	○	○
I prefer video visits to emails	○	○	○	○	○
Video visits reduce my clerical burden	○	○	○	○	○
I would recommend video visits to other providers	○	○	○	○	○
My patients' concerns are addressed by video visits	○	○	○	○	○
The quality of care provided through video visits is nearly equivalent to in-person visits	○	○	○	○	○
My patients have had technical challenges with using video visits	○	○	○	○	○

16. Anything else about video visits that you would like to share? (optional)

© 2021 Neeman E et al. *JAMA Network Open.*

17. Rate yourself on your familiarity with technology: *

	Highly familiar	Somewhat familiar	Neither familiar or unfamiliar	Somewhat unfamiliar	Highly unfamiliar
Email	○	○	○	○	○
Telephone	○	○	○	○	○
Video	○	○	○	○	○
Electronic Medical Record	○	○	○	○	○
General tech savviness	○	○	○	○	○

18. Please indicate any additional telehealth training you may need and enter your contact information if we can contact you re: the training (optional)

19. What is your age? *

- ○ 35 or under
- ○ 36-45
- ○ 46-55
- ○ 56-65
- ○ 66 and older
- ○ Prefer Not to State

© 2021 Neeman E et al. *JAMA Network Open.*

20. What is your gender? *

- ◯ Female
- ◯ Male
- ◯ Prefer Not to State
- ◯ [ ] Other

21. What % of your time is spent on clinical care? *

- ◯ < 60%
- ◯ 60-80%
- ◯ > 80%

22. What was your commute time prior to the pandemic (minutes a day, round trip) *

- ◯ < 30 minutes
- ◯ 30 minutes - 60 minutes
- ◯ >60 minutes - 120 minutes
- ◯ >120 minutes - 180 minutes
- ◯ >180 minutes

© 2021 Neeman E et al. *JAMA Network Open.*

8. Survey tool: Survivorship

Survivorship Survey Telehealth Oncology

Thank you for helping us improve telehealth for our team, our colleagues, and our patients and their families.
Information will be reported out in aggregate, anonymous format only.

Telehealth is defined for the purposes of this study to include email, telephone, and video visits.

* Required

* This form will record your name, please fill your name.

1. What % of your patient encounters are related to telehealth (email, telephone, video)? *

	<25%	26-50%	51-75%	>75%
Before pandemic started	○	○	○	○
During pandemic	○	○	○	○
Expected, after pandemic is over	○	○	○	○

© 2021 Neeman E et al. *JAMA Network Open.*

2. For each of the activities below, please select the method that you believe could be used for a discussion with a patient: *

	Email sufficient, nothing else needed	Phone call sufficient, no video or in person needed	Video visit sufficient, no in person needed	Video visit with shared screen sufficient, no in person needed	In person needed	N/A - I don't preform these types of visits
Introduction to Survivorship Clinic	○	○	○	○	○	○
Discussing scans / test results	○	○	○	○	○	○
Managing symptoms	○	○	○	○	○	○
Managing chronic illnesses	○	○	○	○	○	○
Discussing lifestyle interventions	○	○	○	○	○	○
Discussing recurrence fears	○	○	○	○	○	○
Survivorship planning	○	○	○	○	○	○
Survivorship follow-up	○	○	○	○	○	○
Shared decision making / treatment decisions	○	○	○	○	○	○
Clinical trials enrollment	○	○	○	○	○	○
Clinical trials follow-up	○	○	○	○	○	○

3. Are there other visits that could be performed using telehealth, or visits that could not be performed by telehealth? (optional)

© 2021 Neeman E et al. *JAMA Network Open.*

4. After COVID-19 is no longer an active issue, would you prefer to: *

- ◯ Increase the level of telehealth care
- ◯ Maintain the same level of telehealth care
- ◯ Return to prior levels of telehealth care
- ◯ Use less telehealth than before the pandemic

5. Why (Optional)?

6. What % of patient assessment and care can be managed by the following methods: *

	<25%	25-50%	51-75%	76%-100%
Email	◯	◯	◯	◯
Telephone	◯	◯	◯	◯
Video	◯	◯	◯	◯
In person visit	◯	◯	◯	◯

© 2021 Neeman E et al. *JAMA Network Open.*

7. In your opinion, what is the satisfaction level with telehealth: *

	Very Satisfied	Somewhat satisfied	Neither satisfied nor dissatisfied	Somewhat dissatisfied	Very dissatisfied
Your satisfaction	◯	◯	◯	◯	◯
Colleague satisfaction	◯	◯	◯	◯	◯
Staff satisfaction	◯	◯	◯	◯	◯
Patient satisfaction	◯	◯	◯	◯	◯
Caregiver satisfaction	◯	◯	◯	◯	◯

8. Which of the following benefits of telehealth have you personally experienced? *

	Strong agree	Agree	Neutral	Disagree	Strongly Disagree
Work from home	◯	◯	◯	◯	◯
Reduced commute	◯	◯	◯	◯	◯
Shorter visit time	◯	◯	◯	◯	◯
Stay on time	◯	◯	◯	◯	◯
Autonomy of practice	◯	◯	◯	◯	◯
Flexible hours	◯	◯	◯	◯	◯
Reduced clerical burden	◯	◯	◯	◯	◯

© 2021 Neeman E et al. *JAMA Network Open.*

9. Which of the following benefits of telehealth have your patients or their caregivers experienced? *

	Strongly agree	Agree	Neutral	Disagree	Strongly disagree
Quicker attention to issue or concern	○	○	○	○	○
Less financial burden (copays, parking, etc.)	○	○	○	○	○
More desirable appointment times	○	○	○	○	○
Convenience for caregivers (time off work, etc.)	○	○	○	○	○
More connection with care team or provider	○	○	○	○	○
Patients able to discuss sensitive situations that would be embarrassing or difficult to address in person	○	○	○	○	○

© 2021 Neeman E et al. *JAMA Network Open.*

10. Which negative effects of telehealth have you personally experienced? *

	Strongly agree	Agree	Neutral	Disagree	Strongly disagree
Equipment problems	○	○	○	○	○
Lack of provider training	○	○	○	○	○
Connection problems	○	○	○	○	○
Lack of staff support	○	○	○	○	○
Lack of staff training	○	○	○	○	○
Patient issue should have been answered by another member of the care team	○	○	○	○	○
In person visit required anyway	○	○	○	○	○
Needed physical exam	○	○	○	○	○
Difficulty evaluating performance status	○	○	○	○	○
Difficulty establishing rapport	○	○	○	○	○
Patient suffered an adverse effect that could have been prevented with in-person visit	○	○	○	○	○
Patient not available	○	○	○	○	○

11. What are other benefits or downsides of telemedicine that have not been listed? (optional)

© 2021 Neeman E et al. *JAMA Network Open.*

12. Rate the level of provider-patient connection that each method of communication provides: *

	Strong connection	Weak Connection	No Connection	Worsens Connection
E-mail	○	○	○	○
Telephone	○	○	○	○
Video	○	○	○	○
In person	○	○	○	○

© 2021 Neeman E et al. *JAMA Network Open.*

13. Describe your experience with video visits: *

	Strongly Agree	Agree	Neutral	Disagree	Strongly Disagree
Video visits are easy to implement into my daily clinic work-flow	○	○	○	○	○
I prefer video visits to in-person visits	○	○	○	○	○
I prefer video visits to telephone visits	○	○	○	○	○
I prefer video visits to emails	○	○	○	○	○
Video visits reduce my clerical burden	○	○	○	○	○
I would recommend video visits to other providers	○	○	○	○	○
My patients' concerns are addressed by video visits	○	○	○	○	○
The quality of care provided through video visits is nearly equivalent to in-person visits	○	○	○	○	○
My patients have had technical challenges with using video visits	○	○	○	○	○

14. Anything else about video visits that you would like to share? (optional)

© 2021 Neeman E et al. *JAMA Network Open.*

15. Rate yourself on your familiarity with technology: *

	Highly familiar	Somewhat familiar	Neither familiar or unfamiliar	Somewhat unfamiliar	Highly unfamiliar
Email	○	○	○	○	○
Telephone	○	○	○	○	○
Video	○	○	○	○	○
Electronic Medical Record	○	○	○	○	○
General tech savviness	○	○	○	○	○

16. Please indicate any additional telehealth training you may need and enter your contact information if we can contact you re: the training (optional)

17. What is your age? *

- ○ 35 or under
- ○ 36-45
- ○ 46-55
- ○ 56-65
- ○ 66 and older
- ○ Prefer Not to State

© 2021 Neeman E et al. *JAMA Network Open.*

18. What is your gender? *

- Female
- Male
- Prefer Not to State
- Other

19. What % of your time is spent on clinical care? *

- < 60%
- 60-80%
- > 80%

20. What was your commute time prior to the pandemic (minutes a day, round trip) *

- < 30 minutes
- 30 minutes - 60 minutes
- >60 minutes - 120 minutes
- >120 minutes - 180 minutes
- >180 minutes

© 2021 Neeman E et al. *JAMA Network Open.*
